# Trans-oral miniature X-ray radiation delivery system with endoscopic optical feedback

**DOI:** 10.1007/s11548-017-1601-x

**Published:** 2017-05-09

**Authors:** Axel Boese, Fredrick Johnson, Till Ebert, Ali Mahmoud-Pashazadeh, Christoph Arens, Michael Friebe

**Affiliations:** 1 Chair for Catheter Technologies, Otto-von-Guericke University, Universitätsplatz 2, 39106 Magdeburg, Germany; 2 Clinic of ENT, Otto-von-Guericke University, Leipziger Str. 44, 39120 Magdeburg, Germany

**Keywords:** Trans-oral surgery, Miniaturized X-ray tube, Radiation therapy, ENT, Minimal invasive therapy, Organ-preserving surgery, Tumour bed radiation

## Abstract

**Purpose:**

Surgery, chemo- and/or external radiation therapy are the standard therapy options for the treatment of laryngeal cancer. Trans-oral access for the surgery reduces traumata and hospitalization time. A new trend in treatment is organ-preserving surgery. To avoid regrowth of cancer, this type of surgery can be combined with radiation therapy. Since external radiation includes healthy tissue surrounding the cancerous zone, a local and direct intraoral radiation delivery would be beneficial.

**Methods:**

A general concept for a trans-oral radiation system was designed, based on clinical need identification with a medical user. A miniaturized X-ray tube was used as the radiation source for the intraoperative radiation delivery. To reduce dose distribution on healthy areas, the X-ray source was collimated by a newly designed adjustable shielding system as part of the housing. For direct optical visualization of the radiation zone, a miniature flexible endoscope was integrated into the system. The endoscopic light cone and the field of view were aligned with the zone of the collimated radiation. The intraoperative radiation system was mounted on a semi-automatic medical holder that was combined with a frontal actuator for rotational and translational movement using piezoelectric motors to provide precise placement.

**Results:**

The entire technical set-up was tested in a simulated environment. The shielding of the X-ray source was verified by performing conventional detector-based dose measurements. The delivered dose was estimated by an ionization chamber. The adjustment of the radiation zone was performed by a manual controlling mechanism integrated into the hand piece of the device. An endoscopic fibre was also added to offer visualization and illumination of the radiation zone. The combination of the radiation system with the semi-automatic holder and actuator offered precise and stable positioning of the device in range of micrometres and will allow for future combination with a radiation planning system.

**Conclusions:**

The presented system was designed for radiation therapy of the oral cavity and the larynx. This first set-up tried to cover all clinical aspects that are necessary for a later use in surgery. The miniaturized X-ray tube offers the size and the power for intraoperative radiation therapy. The adjustable shielding system in combination with the holder and actuator provides a precise placement. The visualization of radiation zone allows a targeting and observation of the radiation zone.

## Purpose

**Fig. 1:**
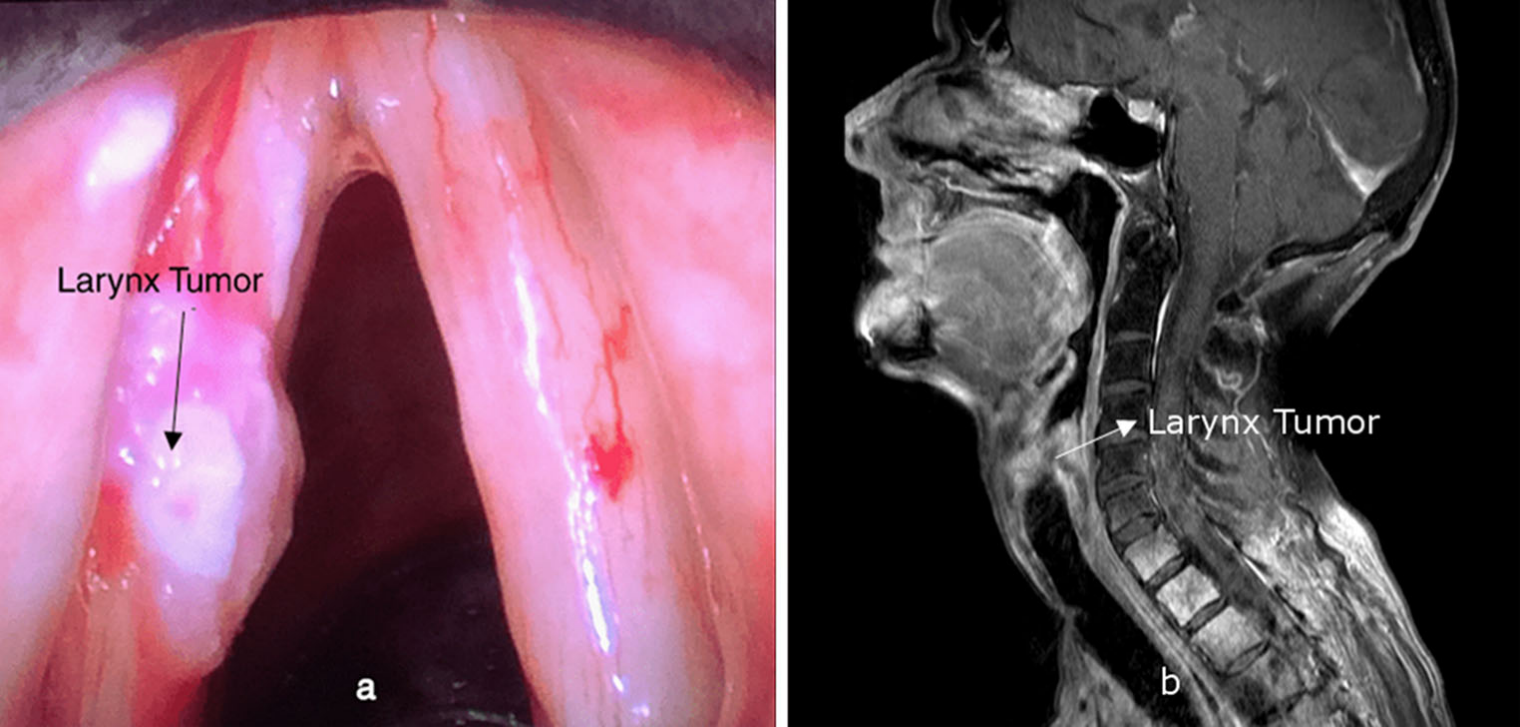
**a** Endoscopic image of a tumour on the vocal fold. **b** MRI Image of a patient showing the larynx tumour (Images: Department of ENT, OVGU Magdeburg)

Larynx carcinoma is a common cancer with about 3800 new cases per year in Germany and about 157,000 new cases per year worldwide. The main cause of the disease is nicotine abuse. The standard therapy includes surgery and/or radio- and chemotherapy, depending on the tumour stage. According to the German “Krebsregister,” the 5-year survival rate currently is 63% [1]. Because of the incidence of the disease, surgical procedures in laryngeal carcinoma belong to the daily routine of the ear nose and throat clinics (ENT). If possible, the interventions are performed via a trans-oral approach in order to avoid an opening of the neck from the outside. In about 20% of the current cases, the opening of the neck is necessary from the outside, since currently no suitable instrument or assistance system is available or surgery cannot be carried out optimally [2].

Instead of total and safe removal of the cancerous tissue, there is nowadays a trend for organ-preserving strategies in surgery that retain speech and swallowing [3]. As little as possible tissue is resected to remove the tumour and ensure the functionality of organs. Current surgical procedures call for a tumour-free margin of about 5 mm [2]. A new strategy is to cut without or with only a very small margin to preserve tissue [4] in selected patients. To reduce the likelihood of tumour recurrence, the surgical resection can be combined with cell killing radiation of the resection zone. External radiation includes damaging the surrounding healthy tissue and risk structures that are proximate [5] and it applies a higher total radiation dose (${\sim }48$ Gy at target structure [6]) to the patient. A local radiation of the tumour bed directly following the resection can reduce that dose to 10–15 Gy [7].

**Fig. 2:**
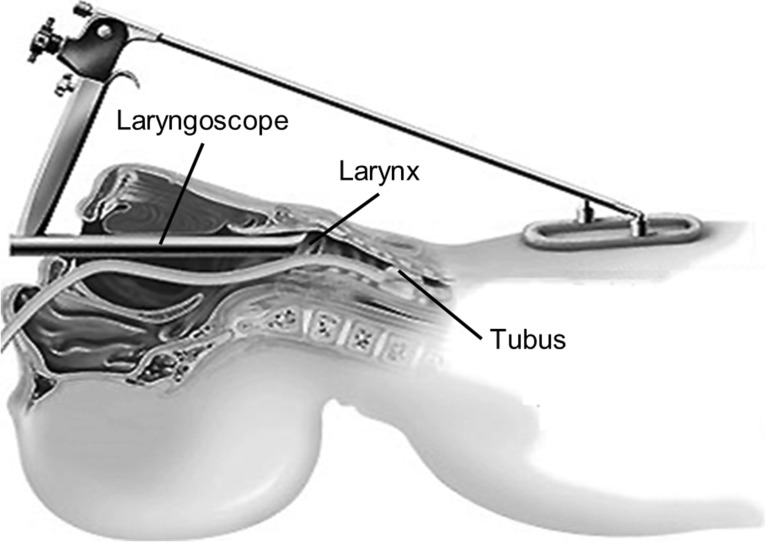
Sketch of the surgery set-up: patient in extended position with laryngoscope and intubation

**Fig. 3:**
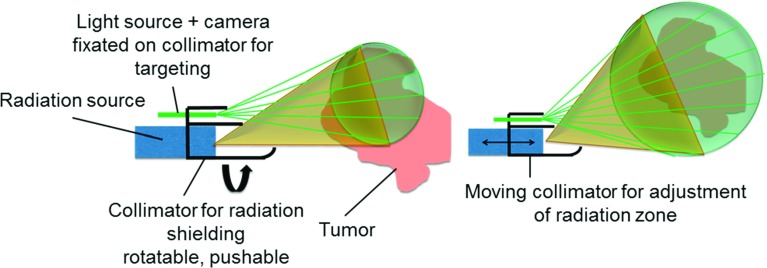
First sketch of combined shielding and targeting mechanism for an intraoperative radiation system; by shifting the collimator, the radiation zone can be adjusted, and the radiation zone is visualized by an overlay of light

The aim of this paper is to present a newly developed prototype system and a treatment strategy based on a miniaturized X-ray source for a local intraoperative radiation therapy after surgery.

## Methods

For the design of the system for trans-oral intraoperative radiation therapy in ENT, we followed a systematically approach based on the evaluation of the clinical needs in cooperation with a medical user [8]. After recording the clinical set-up and workflow, the major functionalities were defined in a general concept. For these functionalities, solutions were designed in short development cycles including user feedback and testing [9]. This approach is further described in the following sections.

### Evaluation of clinical set-up and requirements

Based on medical images, an analysis of the potential target region was performed to define requirements and the clinical integration concept (Fig. 1). Risk structures such as the spinal cord, nerves or the thyroid gland were identified with a clinical expert and will be considered in the later design concept.

Surgeries of the oral cavity and larynx were observed to record the clinical workflow, surgical set-up, equipment and instrumentations used.

The patient typically is placed on the surgical table in the sniffing position. The head is extended, with $35{^{\circ }}$ neck flexion and $15{^{\circ }}$ head extension with a roll underneath the shoulders. The patient is intubated and under general anaesthesia. A laryngoscope with an inner working channel is fed into the endotracheal tube (Fig. 2). Depending on the patient’s anatomy, the working channel of the laryngoscope is between 15 and 30 mm in diameter.

The laryngoscope allows a straight trans-oral access to the larynx. The distance between the entry point of the laryngoscope and the vocal fold as a target region is between 150 and 230 mm in this position. Surgery can be performed under external microscopic or endoscopic imaging with surgical instruments or laser assistance. The resected tumour is collected for pathological examination. After the surgery, the laryngoscope can act as access path for a subsequent local radiation therapy. The tumour size can vary between 10 and 50 mm in diameter [10]. Until now, there is no experience for local direct radiation of the tumour bed after larynx surgery. Based on studies for local radiation on melanoma, breast cancer, sarcoma or colon cancer a cell killing dose of 10–15 Gy can be assumed [7].

**Fig. 4:**
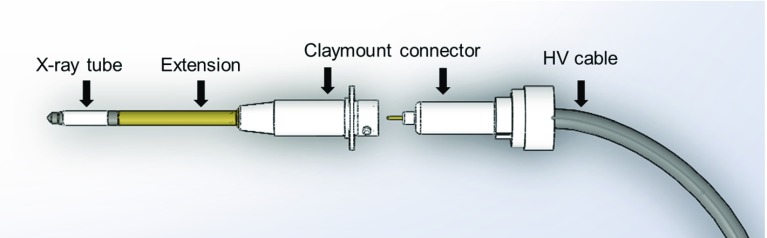
Drawing of CNT X-ray tube with extension and connector

### General conceptual design

Based on the clinical evaluation of surgery, the essential functionalities were defined. For local intraoperative radiation, a small radiation source is needed that can be placed close to the target structure. A shielding or collimation is necessary to avoid radiation of healthy tissue or risk structures. To cover different tumour sizes and shapes, the shielding has to be adjustable. The surgeon must be able to verify the radiation zone. Therefore, this zone has to be marked and observed for visualization (Fig. 3). The radiation system has to be positioned accurately for the duration of treatment. A micro-positioner and stand had to be developed to realize that. The concept was discussed with a clinical expert to meet the requirements for clinical application. The desired parts were defined and designed in a next step.

### X-ray system

A miniaturized X-ray tube was used as the source suitable for intraoperative radiation delivery (CNT-based miniature X-ray tube, KAIST University Korea) [11, 12]. The tube has a diameter of 7 mm and a length of 47 mm. Beside the size, the advantage of CNT-based tube is its lifetime and cold emission radiation. The tube needs no additional cooling as usual in conventional X-ray tubes [11]. It can be operated up to 60 kV. A dose rate of approximate 100 Gy/min can be achieved in permanent operation and optimal conditions [13]. The X-rays are comparatively uniformly distributed in space in an angle of ${\pm }120{^{\circ }}$ measured form central axis of the tube [13].

**Fig. 5:**
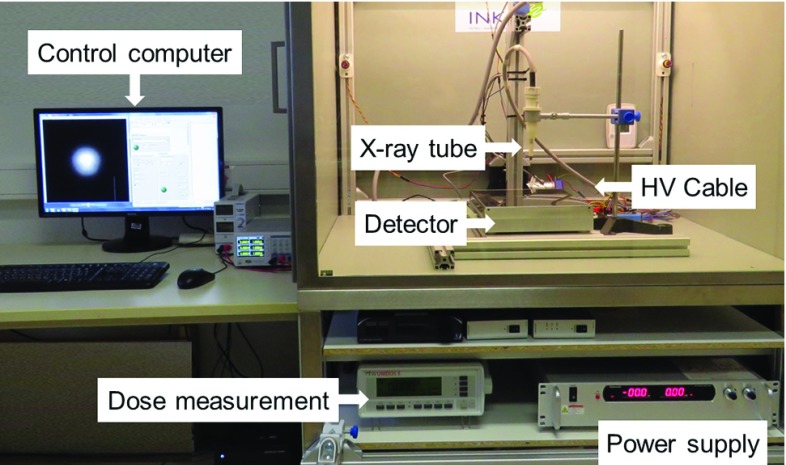
Shielded measure and test set-up for X-ray tubes in the INKA laboratory

**Fig. 6:**
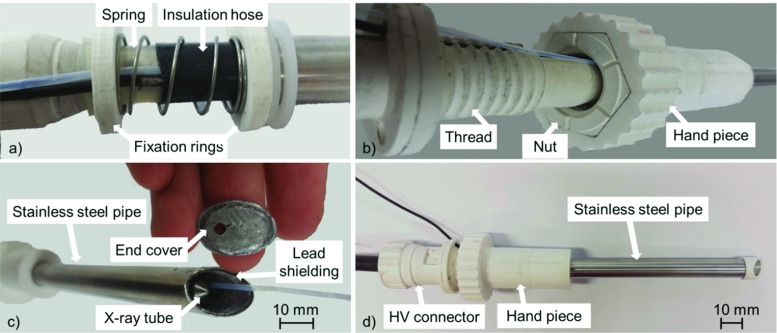
Shielding system for intraoperative radiation: **a**, **b** mechanism for adjustment of the radiation zone, **c** stainless steel pipe with lead shielding, tip of X-ray tube and cover plate, **d** assembly of the shielding system

To achieve a suitable length of the radiation system, the tube is mounted to the high-voltage connector (CA3-type, Claymount, USA) with an extension (Fig. 4). For powering the X-ray tube an external power supply (HVPS AU-60N10, Matsusada, Japan) is used. The X-ray tube is controlled by a computer. The set-up is shown in Fig. 5.

**Fig. 7:**
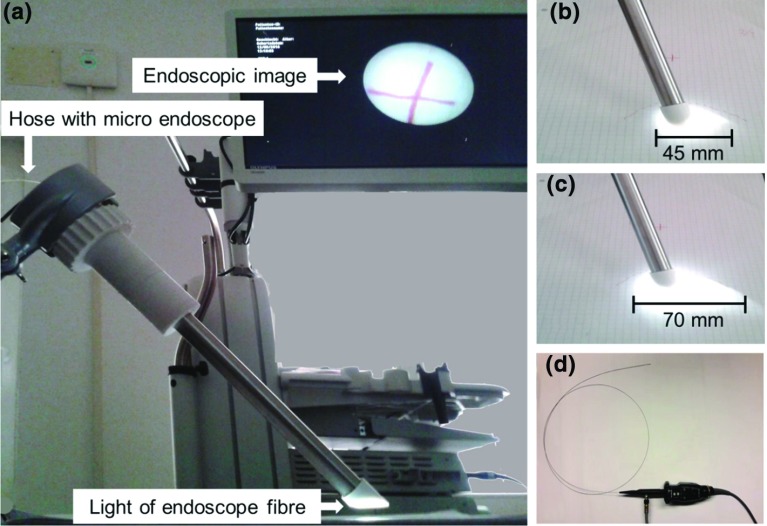
Shielding system combined with endoscopic imaging, **a** test set-up for light alignment showing endoscopic in background and shielding system in foreground, **b**, **c** variation of size of illuminated zone, **d** KALR STORZ prototype endoscopic fibre (Ø 0.5 mm) for illumination and imaging combined with Olympus CH-S190-XZ camera module

### Shielding system

To reduce dose distribution on healthy tissue, the X-ray source is collimated by a newly designed shielding system. The shielding is built up with a biocompatible outer pipe of stainless steel and an inner 1 mm lead layer. On the frontal part, the pipe is cut in a $45{^{\circ }}$ angle. The open end of the pipe is sealed with a cover of 1 mm lead on inside and a stainless steel plate on outside (Fig. 6c). A hole of 3 mm diameter is placed on the frontal part as outlet for radiation. This hole acts as a pinhole collimator. Inside the shielding, the miniature X-ray tube is placed with the extension covered by an insulation hose. The shielding pipe can be moved along the axis of the X-ray tube but is fixed against rotation. A hand piece with an integrated thread allows a precise adjustment of this movement (Fig. 6a, b). The thread is part of the high-voltage connector (CA3-type, Claymount, USA). An adequate screw nut is integrated into the 3D printed hand piece that is rotatable connected with the shielding pipe. By simple rotation of the hand piece the distance between the tip of the X-ray source and the outlet hole of the shielding can be changed. Thus, the shape of the resulting cone beam and therewith the radiation zone can be adjusted similar to the focus mechanism of a torch. An integrated spring assists the movement in the opposite direction (Fig. 6a). The assembly of the shielding system with the integrated X-ray tube is shown in Fig. 6d.

### Endoscopic system

A small plastic hose is integrated in the shielding system (Fig. 6c). A miniature flexible endoscope can be placed through that hose providing optical images of the radiation zone. The tip of the plastic hose is fixed directly on the outlet hole of end cover of the shielding. When adjusting the radiation cone, the visual window of the endoscope follows the radiation zone by deformation of the guiding hose. In a defined depth of introduction of the endoscope, the cone of the endoscopic light is aligned with the zone of radiation (Fig. 7a–c). The flexible endoscope is connected to a standard endoscopic imaging system (EVIS EXERA III + CH-S190-XZ camera module, Olympus, Germany) providing light and video (Fig. 7a, d).

### Holder and actuator

**Fig. 8:**
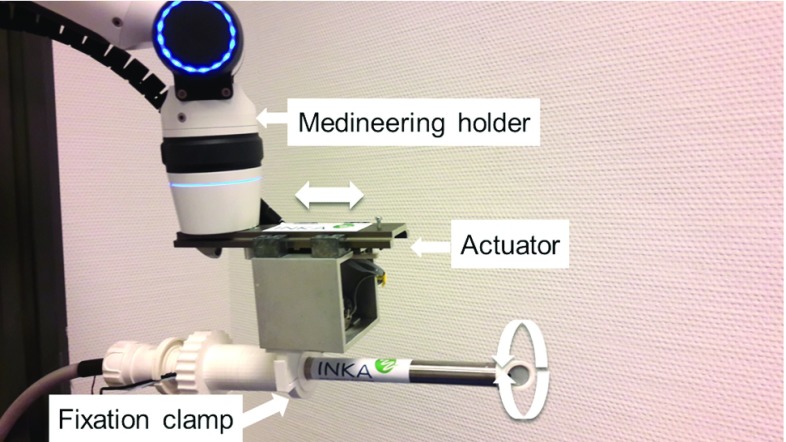
Prototype system for intraoperative radiation therapy combined with actuator and Medineering holder. The actuator allows translational and rotational movement of the system. The 3D printed fixation clamp acts as a fast and easy connection between radiation system and actuator

The shielding system including the miniaturized X-ray tube and endoscopic fibre is combined with a semi-automatic medical holder (Medineering, Germany). The holder allows easy and fast positioning of the radiation system and can be attached fast and easy to standard surgical tables. On the distal tip, an actuator system for precise rotational and translational movement is mounted. The radiation system can be easily attached to the actuator by a fixation clamp. The two integrated piezo motors of the actuator allow a precise movement of the radiation system in the range of micrometres for fine positioning of the device. 90 mm of translational movement and $380{^{\circ }}$ of rotation can be realized. Speed of movement is adjustable. Figure 8 shows the prototype system for intraoperative radiation therapy combined with actuator and Medineering holder. The actuator can be controlled using a foot pedal or via a connected computer. This allows a future combination with radiation planning and automated targeting.

**Fig. 9:**
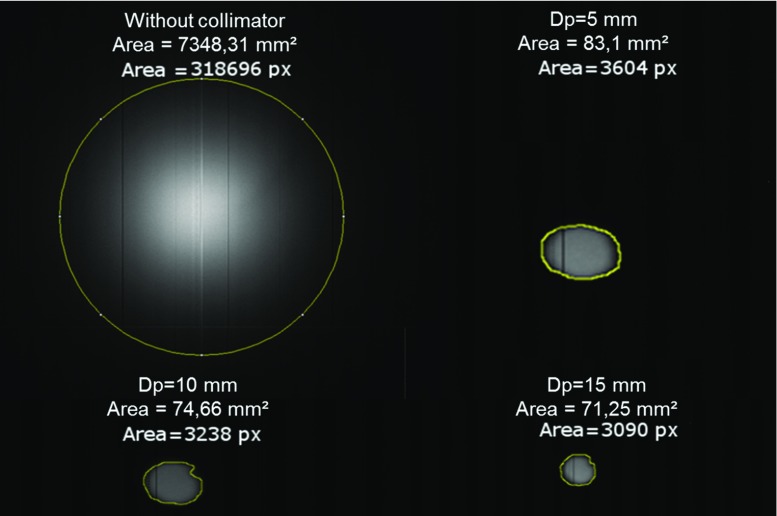
Change of the radiation zone, **a** without shielding **b**–**d** the change with shielding in variant distances (Dp) between outlet hole and X-ray tube, “Area” shows the size of radiation zone and number of pixels that are radiated

### General testing

In order to demonstrate the basic functionality and safety of the system, we performed several tests. The effectiveness of shielding was measured using an ionizing dosimeter chamber (PTW type 34013, PTW, Germany). The distributed dose was recorded without shielding and with shielding on different positions around the X-ray tube.

The expected dose distribution could be measured without shielding and in direct axis of the outlet hole.

To determine the shape of the radiation cone, the shielded X-ray tube was fixed in 10 mm distance above a flat panel detector (Xineos 2222 HS GigE, Teledyne DALSA, the Netherlands). The distance from X-ray tube to pinhole outlet (Dp) was adjusted manually by the hand piece of the shielding system. By increasing the distance, the size of the radiation zone decreases. The radiation zone was imaged with the flat panel detector.

In the same procedure, the overlay of the illuminated area and the field of view of the endoscope were evaluated. The findings were compared to the size of the radiation zone.

**Fig. 10:**
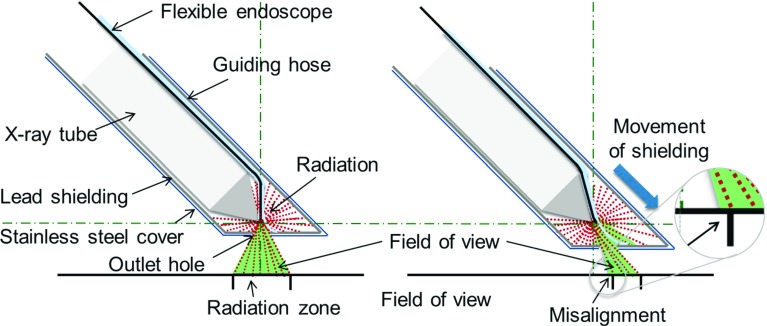
Alignment of light cone and radiation zone, *left* perfect alignment in starting position, *right* misalignment appears after shift of shielding

The system for intraoperative radiation therapy was tested in combination with the Medineering holder and the actuator. The Medineering holder was fixed on a patient table and used for fast positioning of the device. Fine positioning was done by translational and rotational movement of the actuator under image guidance of the integrated fibre endoscope. This interaction was tested on a simple tubular phantom mimicking a laryngoscope and vocal folds as a target structure. Placement was controlled by a foot pedal.

## Results

A system for intraoperative radiation therapy for trans-oral use in ENT interventions was designed in close cooperation with the clinical user. The system was set up as a first prototype. The system was tested on general functionality, handling and safety. The selected X-ray tube offers a clinically acceptable size of only 7 mm diameter and 47 mm length. The temperature of the tip of the X-ray tube can rise up to $60\hbox { }{^{\circ }}\hbox {C}$ when operated. Covered by the shielding system, the resulting outside temperature is approximately $35\hbox { }{^{\circ }}\hbox {C}$ after 20 min of operating. A dose distribution of approximately 2Gy/min in a distance of 20 mm air and 20% duty cycle was measured in the central beam of the tube. When shifting the dosimeter to the side and out of the radiation cone of the outlet hole, no dose distribution could be detected. According to the literature [7], for cell killing effect in direct radiation, approximately 10–15 Gy can be assumed for treatment of tumour bed after resection. Therefore, a radiation time of approximately 5–8 min is necessary.

The newly designed shielding system safely covers the radiation. The adjustment mechanism enables a reproducible variation of the radiation zone. An adjustment of radiation zone of 45–70 mm diameter was demonstrated.

Figure 9 shows the radiation zone without shielding and the change with shielding in variant distances (Dp) between outlet hole and X-ray tube.

The shielding system for intraoperative radiation provides an overlay of an illuminated area to visualize the radiation zone.

As expected, there is a mismatch in the overlapping of radiation and visualization. This failure varies depending on the distance between tip of the X-ray tube and outlet hole. In starting position, light cone and radiation cone are aligned perfectly. When increasing the distance between X-ray tube and outlet hole, a shift between illuminated area and radiation zone occurs (Fig. 10).

The shielding system for intraoperative radiation was combined with a semi active holder provided by Medineering. This allows a rough but fast positioning of the device. The fine positioning can be achieved using the translational and rotational movement of the actuator under image guidance of the integrated fibre endoscope.

The use of Medineering holder offered fast integration in a clinical set-up. The attachment of the shielding system with the integrated X-ray tube was easy realized with a catching clamp. Fine placement with the actuator is possible in 0.001 mm steps of translational movement and $0.1{^{\circ }}$ steps of rotation. By using the foot pedal, the surgeon can assist the placement with his hands and observe the procedure on screen of the endoscopic system.

## Discussion

The presented system is a first step to establish intraoperative radiation therapy in trans-oral treatments. Existing radiation systems are made for external radiation or are oversized for trans-oral applications. It was shown that a set-up including a radiation source, shielding and visualization is technical feasible in an adequate size for trans-oral use.

In comparison with boost therapy with radionuclides, a radiation system based on X-ray is much more comfortable for the clinical user. The dose delivered to the tissue decreases with an increase of penetration depth. At 60 kV, the attenuation loss is approx. 50% in 2 cm depth [14]. After treatment, the radiation can be switched off after treatment.

The X-ray-tube can deliver adequate dose for cell killing in reasonable time of 5–8 min. The reliability of the X-ray tube and the variance between different tubes are still a big issue. The manufacturing process of the tubes and especially the sealing of the inner vacuum have to be optimized to ensure constant quality. Thus, the dose rates published in the literature by other groups could not be achieved [13]. Higher efficiency can lead to reduction of radiation time.

The outlet hole in the shielding used in the described testing’s was 3 mm leading to an adjustable radiation zone of Ø 45–70 mm. This has to be reduced for more precise targeting and to meet the size of tumour in the larynx (Ø $\sim$ 10–50 mm) [10]. A smaller radiation zone also offers a better chance to spare healthy tissue areas on irregularly shaped tumours.

The alignment of the field of view, the illumination and the radiation zone should be improved further. The shift of radiation zone as demonstrated in Fig. 10 is predictable. An equipment of the endoscopic fibre with a specially adjusted lens or prism could reduce this failure.

We presented just a first prototype of a shielded system for local trans-oral radiation but with a major focus on clinical usability and integration. The described set-up has the potential for easy installation in a standard surgical theatre without the need of fundamental changes and can be included in the clinical workflow of surgery.

The single parts of this conceptual design still need further improvement in handling, dimension, geometrical shape and technical reliability for clinical application. These works have to be realized in a dedicated design process for medical devices.

Future works should combine the system with image registration to prepare for integration of a radiation planning. Additionally radiation tests on living cells should be performed to develop adequate radiation protocols and prove lethal dose. Also the shape and size of radiation zone have to be verified in further experiments.
